# Congenital giant fibroepithelial polyp of the scrotum in an infant: the first case report from China

**DOI:** 10.3389/fped.2023.1191983

**Published:** 2023-07-10

**Authors:** Chenghao Zhanghuang, Yu Hang, Fengming Ji, Na Long, Zhigang Yao, Li Li, Zhen Yang, Haoyu Tang, Kun Zhang, Chengchuang Wu, Yucheng Xie, Bing Yan

**Affiliations:** ^1^ Department of Urology, Kunming Children’s Hospital (Children’s Hospital Affiliated to Kunming Medical University), Kunming, China; ^2^ Yunnan Key Laboratory of Children’s Major Disease Research, Yunnan Province Clinical Research Center for Children’s Health and Disease, Kunming, China; ^3^ Department of Special Needs Ward, Kunming Children’s Hospital (Children’s Hospital Affiliated to Kunming Medical University), Kunming, China; ^4^ Department of Oncology, Yunnan Children Solid Tumor Treatment Center, Kunming Children’s Hospital (Children’s Hospital Affiliated to Kunming Medical University), Kunming, China; ^5^ Department of Pathology, Kunming Children’s Hospital (Children’s Hospital Affiliated to Kunming Medical University), Kunming, China

**Keywords:** childhood, fibroepithelial, male, pathology, scrotum, case report

## Abstract

Giant fibroepithelial polyp (FP) of the scrotum in infants is a rare disease. We reported the first case of FP in China. The child was only 9 months and 12 days old and was admitted to the hospital due to rapid growth and rupture of the scrotal mass. The patient underwent scrotal exploration under general anesthesia, and the mass was cystic-solid with clear boundaries. The tumor did not invade the sarcolemma of the scrotum and testicular tissue. The intraoperative pathological frozen section tended to be benign, and the scrotum's tumor and subcutaneous pedicle tissue were removed entirely after 0.5 cm from the boundary of the mass. The operation was successful. The mass was confirmed as FP by postoperative pathology. 6 months after the operation, the incision healed well without recurrence. This case report has a detailed diagnosis and treatment process and adequate examination results. It can provide a reference for diagnosing and treating FP in infants and reduce the risk of misdiagnosis and mistreatment.

## Background

Fibroepithelial polyps (FP), called soft fibromas, are a kind of pedunculated benign tumor. It is common in middle-aged or older adults (1), especially postmenopausal women, and can also be seen in pregnant women (2). Congenital FP of scrotal origin in infants is particularly rare. So far, only scattered reports have been reported. In 2008, Hyun CH and other Korean scholars reported a case of an 18-month-old male child with FP located at the penoscrotal junction. The mass was soft, dark red, non-tender, and crest-like (3). In January 2021, Kubelis-Lopez et al. in Mexico reported a 3-month-old case of giant FP in the scrotum, which was completely resected and obtained a good prognosis (4). When an infant presents with a vulvar mass, a detailed history, physical examination, and pathological examination are essential (5). At the same time, it is also crucial to evaluate the vascular invasion level (6).

Here, we report a case of a 9-month-old boy with a rapidly expanding mass in the scrotum that ruptured and was completely removed by surgery. Postoperative pathology confirmed that the child was a giant FP of the scrotum, and there was no recurrence after 6 months of reexamination.

## Case report

**Figure 1:**
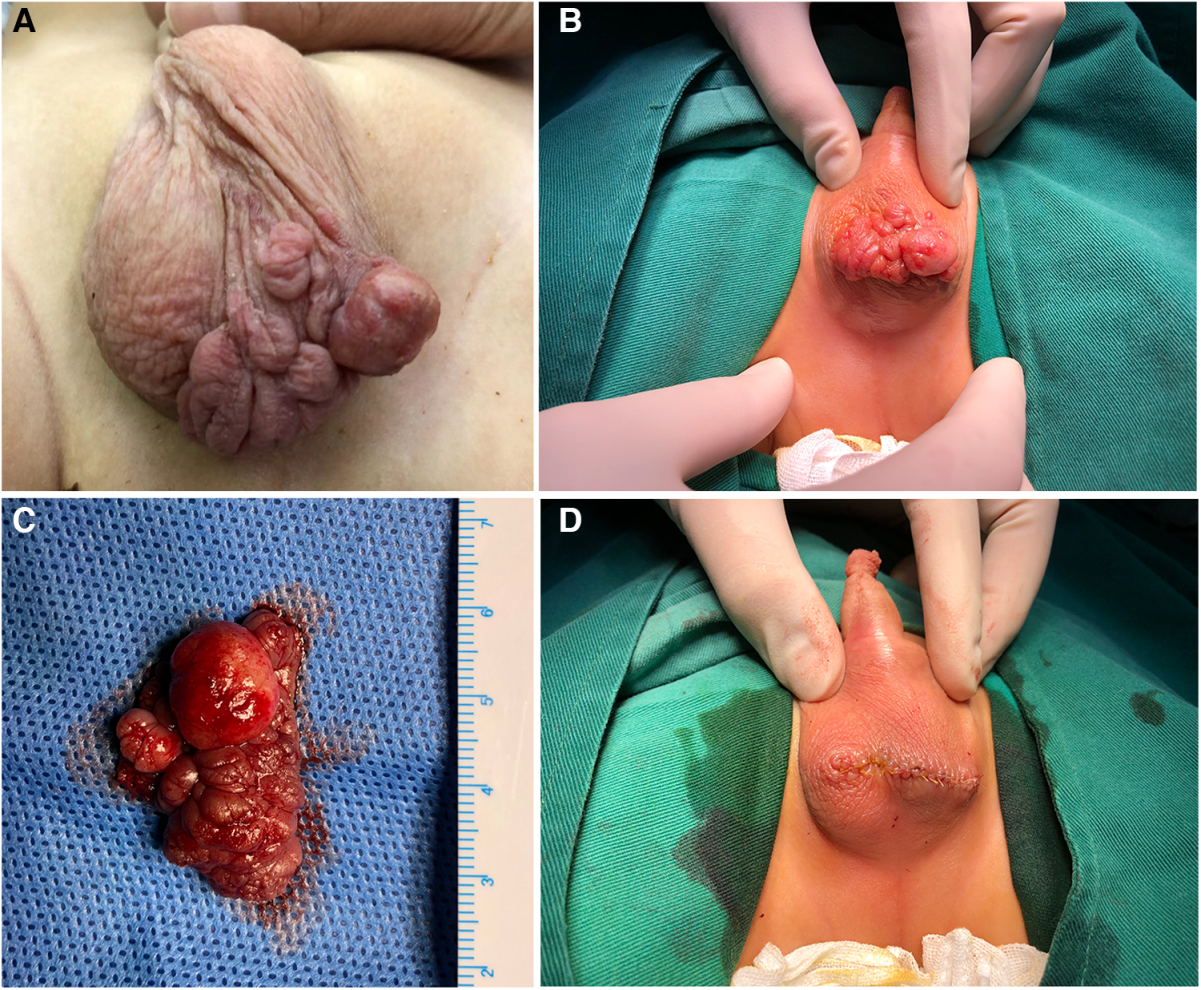
Preoperative and intraoperative findings of specialized physical examination; (**A,B**) giant scrotal mass growing in cauliflower-like pattern with clear boundary with normal scrotum; (**C**) gross picture after complete resection of the mass during operation; (**D**) pictures of incision suture after mass resection.

**Figure 2:**
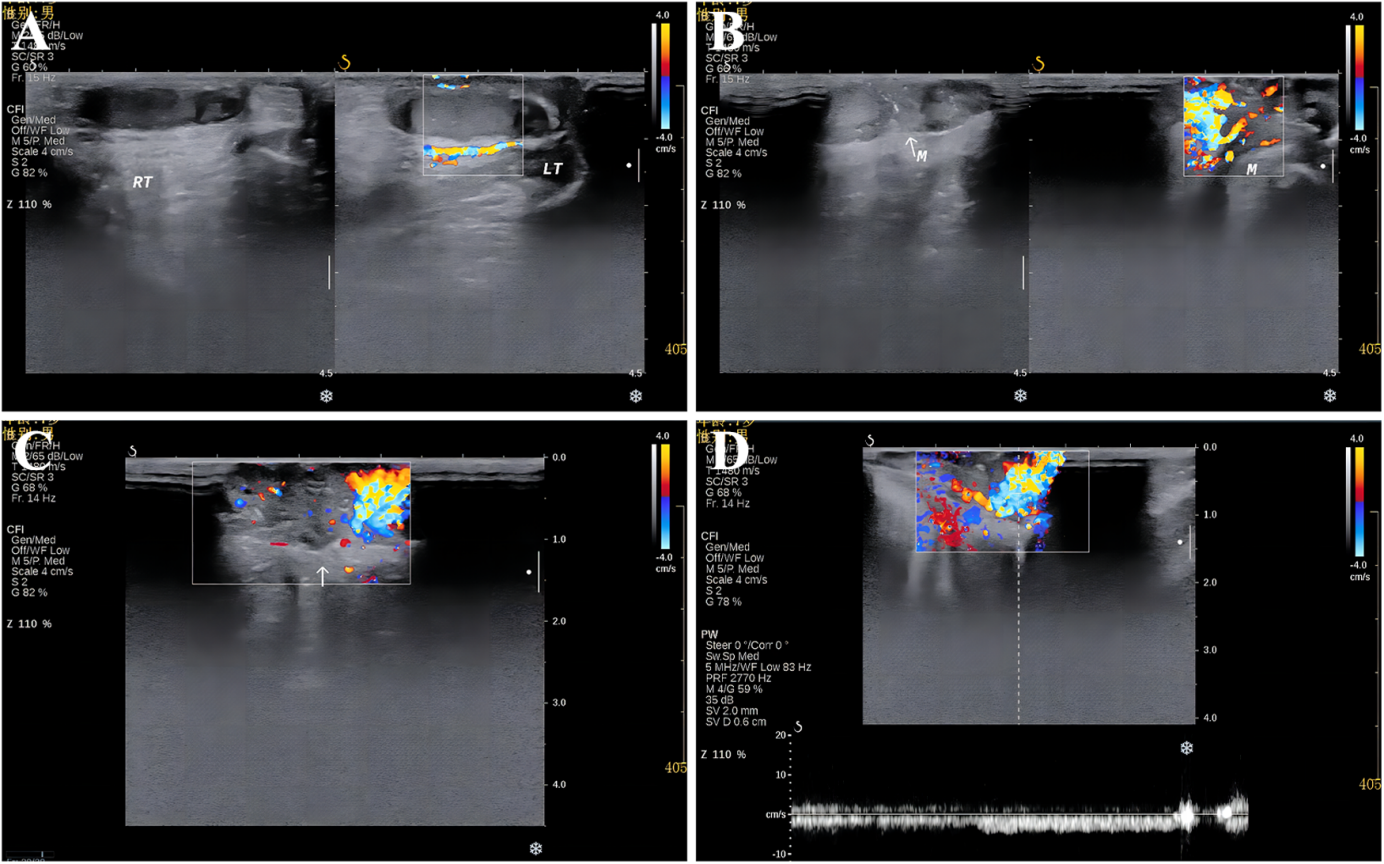
Preoperative colour Doppler ultrasonography; (**A**) bilateral testicular colour Doppler ultrasonography; (**B,C**) giant scrotal mass detected by CDFI; (**D**) findings on PD examination of the giant scrotal mass.

At birth, the child was found by his parents to have a light purple mass in the left scrotum, about 0.5 cm × 0.5 cm in size, protruding from the scrotal skin, with clear boundaries, tough texture, and no tenderness. The patient had no fever and was in good general condition. The family members of the children ignored it and did not go to medical institutions for treatment. The mass enlarged rapidly with age and covered most of the left scrotum. One week ago, the mass ruptured when his family changed the diaper. The scrotal mass of the patient was ruptured from a “mass” to a “cock's comb.” The ulcerated surface resembled new granulation tissue with a small amount of oozing blood. He went to our department for further diagnosis and treatment. Since developing the disease, the child has had good spirits, a good diet, regular urination and defecation, and weight gain. After admission, the relevant examinations were performed. The specialized physical examination showed a mass in the left scrotum with a size range of 6.5 cm × 5.5 cm, which was cauliflower-like growth and scattered with a broad base. There was no obvious blood oozing, redness, swelling, or tenderness, and a clear boundary with normal scrotal skin (Figure 1A). Preoperative ultrasound showed a mass of 6.2 cm × 5.0 cm × 2.0 cm, irregular shape, “cauliflower-like” protruding from the scrotum wall, hypoechoic inside, and clustered hyperechoic spots. Color Doppler flow imaging (CDFI) showed abundant bar-like blood flow signals. Pulsed Doppler (PD) displays the spectrum of arteries and veins; the arterial flow rate was 14.9 m/s, the blood flow resistance index (RI) was 0.43, and the venous flow rate was 5.3 cm/s. The ultrasonographic findings were as follows: (1) The image of a solid mass in the scrotum wall (hemangioma? Non-exclusive malignant lesions). (2) Bilateral testes and epididymis were normal (Figure 2). Combined with the above examination results, because the boundary of the mass was clear, ultrasound showed no adhesion between the base of the mass and the tissue of the bilateral testes, so the possibility of a benign epidermal tumor was considered. The initial preoperative diagnosis was a “space-occupying lesion of the scrotum of unknown origin (hemangioma?).”

**Figure 3:**
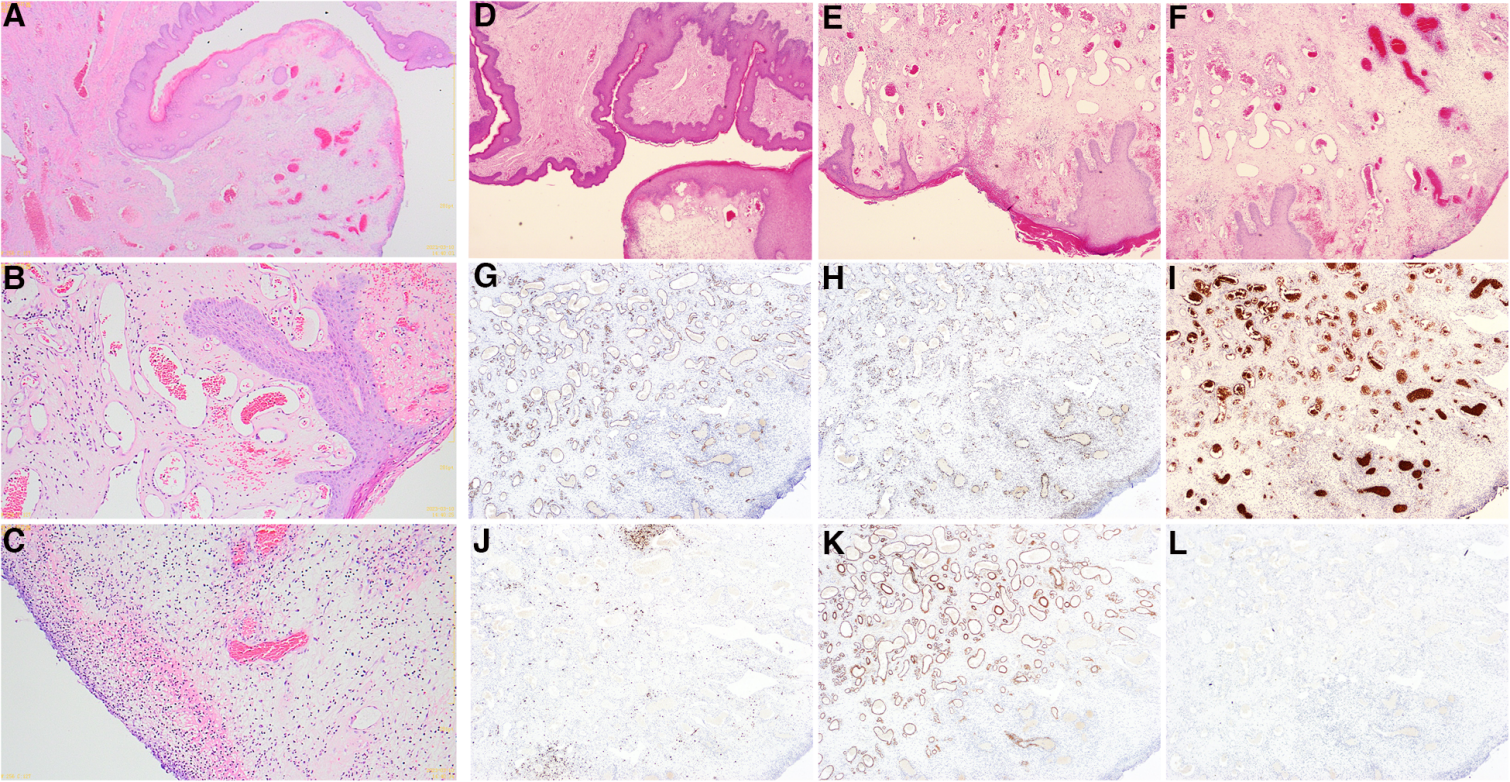
Intraoperative frozen pathology and postoperative pathological examination pictures; (**A–C**) intraoperative frozen HE staining findings (**A**: 10X, **B,C**: 40X); (**D–F**) a postoperative pathological examination of HE staining (**D**: 10X, **E,F**: 40X); (**G–L**) immunohistochemical findings of postoperative pathological examination (**G**: CD34, **H**: ERG, **I**: MSA, **J**: S-100, **K**: SMA, **L**: SOX-10) (**G–L**: 40X).

Scrotal tumor resection was performed under general anesthesia after excluding surgical contraindications. Intraoperative frozen pathology showed that the tumor tended to be benign (Figures 3A–C). After entirely free of the mass and scrotal subcutaneous pedicle tissue 0.5 cm from the boundary of the mass, the tumor was removed completely (Figures 1B–D). Postoperative pathological examination showed that the surface of the mass was covered with stratified squamous epithelium, with partial epithelial loss, and pus on the surface, beneath which were proliferated fibers, blood vessels, smooth muscle, and fat, accompanied by a large number of acute and chronic inflammatory cell infiltration (Figures 3D–F). Immunohistochemistry showed CD34 (+), ERG (+), muscle-specific actin (MSA) (−), S-100 (−), smooth muscle actin (SMA) (+), and SOX-10 (−) (Figures 3G–L). The pathological diagnosis was a fibroepithelial polyp of the scrotum with an ulcer. The patient was discharged 3 days after the dressing change, and the incision healed well 6 months after the operation without recurrence.

## Discussion

FP mainly occurs in postmenopausal women and is considered to be closely related to estrogen levels and the occurrence of polycystic ovaries (7). Previous studies have shown that FP may be related to metabolic syndrome such as diabetes because the incidence of FP is significantly increased in people with elevated BMI and high-density lipoprotein (8). In addition, some scholars found a significant positive correlation between trauma and the occurrence of FP (9). However, the relationship between human papillomavirus (HPV) and FP remains controversial. Gupta et al. found high expression of HPV6/11 in patients with FP (10). On the contrary, a 2012 Iran study showed no significant correlation between HPV and FP occurrence (11). In conclusion, there is no unified standard for the etiology of FP. Congenital FP in infants is rare in clinical practice, there are few studies at present, and the etiology is unknown.

FP usually occurs in the face and neck, axilla, and groin regions. A few are located in the vulva (12), bladder neck (13), ureter (14), and oral cavity (15). Occasionally it occurs in the penis (16) and anus (17). There are only a few reports of giant FP in the scrotum, and the relevant important examinations, such as immunohistochemistry, have not been completed. FP's diagnosis and differential diagnosis cannot be determined (3, 4). Based on this, our case report is the first from China with a detailed diagnosis, treatment, and sufficient examination results. It can provide a reference for diagnosing and treating scrotal FP and reduce the risk of misdiagnosis and delayed treatment.

There are few studies on the pathophysiology and susceptibility of FP. El Safoury et al. analyzed skin sections from 15 patients with skin vegetations (TS) using enzyme-linked immunosorbent assay and reverse transcriptase polymerase chain reaction and showed that mast cells, tumor necrosis factor-α (TNF-α), and TNF-related apoptosis-inducing ligand (TRAIL) might play an important role in ST patients (18). Farag et al. also collected 40 TS patients and 20 control patients in Egypt for gene polymorphism analysis and proved that insulin-like growth factor-1 (IGF-1) plays a key role in the susceptibility to FP (19). In addition, a report published in 2004 suggested that down-regulation of tuberin and hamartoma expression may benefit fibroblast proliferation or promote collagen production, leading to the occurrence and development of FP (20).

Typical FP is characterized by the formation of pedunculated polypoid nodules protruding from the skin surface. According to the morphological characteristics, it can be divided into three types: (1) exanthematous soft fibromas: multiple small papules, usually occurring on the neck; (2) filamentous soft fibroma: filamentous growth of myxoid processes; (3) pedicle type soft fibroma: more common in the lower part of the trunk and inguinal region, with pedunculated polypoid processes, soft, often more than 1 cm in diameter. Giant FP refers to FP larger than 5 cm in diameter, which is rare in a clinic and mainly occurs in adults (21). According to the above classification, this case of FP is a giant pedicled soft fibroma.

Color doppler ultrasound and other imaging examinations have some value in diagnosing FP. FP can appear as a hypoechoic mass with clusters of hyperechoic spots on ultrasound. Abundant rod-like blood flow signals can be seen under CDFI (3). However, due to the rupture and other reasons, internal vascular proliferation is obvious, and it is difficult to effectively identify hemangioma by ultrasound, which increases the misdiagnosis rate (6). The primary cutaneous FP is superficial in location, and the role of computed tomography (CT) and magnetic resonance imaging (MRI) is limited (22). At the same time, some studies have shown that CT has particular value in the diagnosis of FP of the ureter and bladder (23). MRI plays a certain role in the diagnosis of FP in the epidermis. T1WI is an equal or low signal, T2WI is a low signal, and delayed enhancement after an enhanced scan is early (24). However, because children cannot cooperate, it is not recommended as a routine preoperative examination for children with FP (22).

The diagnosis of FP requires pathological examination. The typical gross feature of FP is a pedunculated polypoid mass protruding from the skin surface, with a gray-white or gray-yellow cut surface and soft texture (3). Microscopically, the tumors were covered with squamous epithelium, with proliferating fibers and blood vessels under the epithelium, often accompanied by myxoid degeneration in the stroma, fatty tissue, and occasional inflammatory cell infiltration (4). Most FP can be diagnosed by HE staining. However, we recommend that immunohistochemical examination should be performed to avoid misdiagnosis.

FP should be differentiated from Dermatofibrosarcoma protuberans (DFSP) and Pleomorphic fibroma of the skin (PFS). Microscopically, DFSP comprised all peripheral nerve components, including Schwann cells and fibroblasts. Most of the nuclei were slender, wavy, and serpentine. The positive expression of S-100 and SOX-10 in immunohistochemistry can confirm the diagnosis of DFSP. In contrast, however, FP was negative for both measures. In PFS, the tumor comprised a small number of spindle cells and abundant collagen fiber bundles, with characteristic multinucleated giant cells and many pleomorphic cells. Immunohistochemically, MSA and CD34 were positive and could be distinguished from FP. In this case, the immunohistochemical S-100, S0X-10, and MSA were negative, which provided a solid basis for the differential diagnosis of FP.

Since FP is a benign tumor, local surgical resection is the primary means of clinical treatment. However, paying attention to the complete separation and resection of the tumor and the pedicle tissue during the operation is necessary, which can effectively avoid recurrence and obtain a satisfactory cure rate (25). However, there are occasional reports of secondary squamous and basal cell carcinoma at the site of FP resection (26). Clinicians should ensure the length and frequency of postoperative follow-up to avoid the recurrence of FP and secondary malignant tumors.

## Conclusion

Congenital giant FP of the scrotum in infants is clinically rare, and our center has performed the first case report from China. The lack of obvious clinical symptoms can easily cause guardian neglect, thus delaying treatment. The diagnosis of FP requires a combination of pathological HE staining and immunohistochemical results. The disease is a benign tumor with a good prognosis. However, attention should be paid to monitoring during clinical diagnosis and treatment to reduce the risk of recurrence and malignant transformation.

## Data Availability

The original contributions presented in the study are included in the article, further inquiries can be directed to the corresponding authors.
